# Mitochondria as a possible target for nicotine action

**DOI:** 10.1007/s10863-019-09800-z

**Published:** 2019-06-13

**Authors:** Dominika Malińska, Mariusz R. Więckowski, Bernadeta Michalska, Karolina Drabik, Monika Prill, Paulina Patalas-Krawczyk, Jarosław Walczak, Jędrzej Szymański, Carole Mathis, Marco Van der Toorn, Karsta Luettich, Julia Hoeng, Manuel C. Peitsch, Jerzy Duszyński, Joanna Szczepanowska

**Affiliations:** 1 Nencki Institute of Experimental Biology, Polish Academy of Sciences, 3 Pasteur Street, 02-093 Warsaw, Poland; 2 PMI R&D, Philip Morris Products S.A. (part of Philip Morris International group of companies), Quai Jeanrenaud 5, 2000 Neuchâtel, Switzerland

**Keywords:** adaptation, mitochondria, nicotine, oxidative stress

## Abstract

Mitochondria are multifunctional and dynamic organelles deeply integrated into cellular physiology and metabolism. Disturbances in mitochondrial function are involved in several disorders such as neurodegeneration, cardiovascular diseases, metabolic diseases, and also in the aging process. Nicotine is a natural alkaloid present in the tobacco plant which has been well studied as a constituent of cigarette smoke. It has also been reported to influence mitochondrial function both *in vitro* and *in vivo*. This review presents a comprehensive overview of the present knowledge of nicotine action on mitochondrial function. Observed effects of nicotine exposure on the mitochondrial respiratory chain, oxidative stress, calcium homeostasis, mitochondrial dynamics, biogenesis, and mitophagy are discussed, considering the context of the experimental design. The potential action of nicotine on cellular adaptation and cell survival is also examined through its interaction with mitochondria. Although a large number of studies have demonstrated the impact of nicotine on various mitochondrial activities, elucidating its mechanism of action requires further investigation.

## Introduction

### Nicotine

Nicotine is a natural alkaloid discovered in *Nicotiana tabacum* by Posselt and Reimann in 1828 and was first synthesized by Pictet and Rotschy in 1904. It is produced in the roots of the plant and accumulates in the leaves and is also found in smaller amounts in other members of the *Solanaceae* family of flowering plants such as potatoes, tomatoes, eggplants and chilli peppers (Saitoh et al. 1985). Nicotine is a small molecule composed of pyridine and a pyrrolidine ring that exists in two enantiomeric forms: (i) levorotatory ([-]-nicotine), its natural form, and (ii) dextrorotatory ([+]-nicotine). Nicotine constitutes 0.6% - 3% of dry weight of tobacco and is the primary addictive component of tobacco smoke and other nicotine-containing products. The time course of nicotine exposure and its impact on the brain and other body organs highly varies if it is delivered through the use of tobacco products or nicotine replacement therapy such as transdermal patches or gum (Hukkanen et al. 2005). Depending on the routes of administration, nicotine can be absorbed through the wall lining of the alveoli in the lungs, through the mucous membranes of the nose or mouth, through the digestive tract or through the skin. It circulates then via the bloodstream throughout the body and easily crosses the blood-brain barrier to reach the central nervous system. It is thus considered a systemic drug. When a cigarette is smoked, nicotine is detectable in the brain in as little as seven seconds after smoke inhalation, and it reaches its maximum level 4-5 minutes later (Berridge et al. 2010). As nicotine is a ligand for nicotinic acetylcholine receptors (nAChRs) (Powledge 2004), it binds to nAChRs expressed in brain tissues with high affinity. This receptor binding capacity is even increased in smokers compared to nonsmokers (Perry et al. 1999) due to a higher level of nAChR expression in smokers’ brains (50% higher than in those of nonsmokers) (Benwell and Balfour 1985). Nicotine absorption through cell membranes is possible due to its amphiphilic nature, but it is also dependent on environmental pH (nicotine pKa = 7.9). At low pH, nicotine does not readily cross membranes, but in the blood, where the physiological pH is around 7.4, 31% of nicotine is non-ionized and can easily pass through cell membranes (Le Houezec 2003). In humans, nicotine has a metabolic half-life of two hours. A small part of it (10-20%) is directly excreted in urine. The other part is metabolized by cytochrome P450 2A6 (CYP2A6), mainly in the liver (and to a lesser extent also in lung, kidney, nasal mucosa and brain). The primary metabolite of nicotine is cotinine (70-80%), with small fractions of the compound being converted to nicotine-N-oxide (4%), nornicotine and 4-(3-pyridyl)-4-hydroxybutanoic acid, or being conjugated to nicotine glucuronide (Benowitz et al. 2009; Hukkanen et al. 2005).

Nicotine exposure impacts numerous organ systems (neurological, neuromuscular, cardiovascular, respiratory, immunological and gastrointestinal), and the majority of nicotine effects are mediated by the activation of nAChRs in a wide variety of neuronal and non-neuronal tissues (Lee and Fariss 2017). Of note, nicotine effects are influenced by the presence of different types of nAChRs and by how these receptors are regulated and functioning (Lam et al. 2016; Marks et al. 1987; Renda and Nashmi 2014). Indeed, in addition to the direct pharmacological activation of nAChRs, nicotine also acts as a pharmacological chaperone of nAChRs, favouring their assembly by modulating the expression level of nicotinic acetylcholine receptor regulator chaperone (NACHO), an important regulator of nAChR maturation and surface expression (Wichern et al. 2017).

Chronic nicotine exposure has been linked to various health effects described in human as well as in animal model studies. The safety of nicotine use is still under scrutiny, especially with the large number of people exposed daily to it in an attempt to quit smoking (Lee and Fariss 2017). The United Kingdom’s National Institute for Health and Care Excellence concluded in 2013 that “evidence is available from studies with up to 5-year follow-up which suggests that ‘pure’ nicotine, in the form available in nicotine replacement therapy (NRT) products, does not pose a significant health risk” (National Institute for Health and Care Excellence 2013). This statement is still under debate and does not consider nicotine’s potential adverse effects during development (England et al. 2017). In addition, a recent review by Haussmann and Fariss evaluated available epidemiological and *in vivo* evidences to determine whether nicotine has carcinogenic properties (Haussmann and Fariss 2016). They concluded that the evidence was inadequate to derive a clear answer (Haussmann and Fariss 2016).

Various scientific reports also suggested that nicotine could be beneficial. Indeed, there is evidence for nicotine having anti-inflammatory and anti-apoptotic properties (Benowitz 2010; Copeland Jr et al. 2007; de Jonge and Ulloa 2007; Picciotto et al. 2008; Quik et al. 2012). In particular, various studies reported a beneficial effect of nicotine exposure in obesity and ulcerative colitis, involving the activation of a cholinergic pathway through α7 nAChR on immune cells (Lakhan and Kirchgessner 2011). A suppressive effect on the expression of pro-inflammatory cytokines was described in nicotine-exposed cells through modulation of the NF-κB signalling pathway (Cui and Li 2010). Clinical trials and animal studies also indicated that nicotine exposure could improve cognitive function (Barr et al. 2008) and that it may provide partial relief of symptoms in patients suffering from neurodegenerative diseases (Veljkovic et al. 2018). A growing body of evidence suggests that mitochondria play a crucial role in the pathophysiology of inflammatory bowel disease (IBD) (Lykhmus et al. 2017) and several neurodegenerative diseases, including Alzheimer’s, Huntington’s, Parkinson’s diseases and amyotrophic lateral sclerosis (Lykhmus et al. 2017; Uspenska et al. 2018).

Due to the fact that nicotine has been reported to influence mitochondrial function both *in vitro* and *in vivo* and the precise mechanisms underlying the effects of nicotine on mitochondrial function remain largely unknown, this review will focus on the primary action of nicotine on mitochondrial physiology at the cellular and organism level.

### Mitochondria

Mitochondria are multifunctional, dynamic organelles that are found in every human cell except mature erythrocytes and are critical to its normal function. Mitochondria have a double membrane which separates them into four distinct compartments: the outer mitochondrial membrane (OMM), intermembrane space (IS), inner mitochondrial membrane (IMM) and mitochondrial matrix. The OMM separating mitochondria from the cytosol is easily permeable for most ions and small molecules, contrary to the IMM which is highly impermeable. The IMM is folded into structures called cristae, where the assembly of the respiratory chain enzyme complexes and ATP synthase takes place. Mitochondria are equipped with their own DNA (mtDNA) which contains 37 genes encoding critical subunits of the respiratory chain enzymes, ribosomal RNAs and transfer RNAs. Most mitochondrial proteins are encoded by the nuclear genome, translated in the cytoplasm and transported to mitochondria (Graff et al. 1999; Lee and Han 2017).

Mitochondria are called the power houses of the cell, because one of their most important functions is the production of ATP by oxidative phosphorylation (OXPHOS). The OXPHOS system consists of four enzyme complexes of the electron transport chain (ETC): NADH:ubiquinone oxido-reductase or NADH dehydrogenase (complex I), succinate dehydrogenase (complex II), coenzyme Q: cytochrome c – oxido-reductase or cytochrome bc_1_ (complex III), cytochrome c oxidase (complex IV) and ATP synthase (also called complex V). Electrons from NADH and succinate enter the ETC through complex I and II and are transferred to coenzyme Q, complex III, cytochrome c, complex IV and finally to molecular oxygen which is reduced to water. The energy released by electrons flowing through complexes I, III and IV allows for pumping protons out of the matrix. The resulting proton-motive force combined with a pH gradient (Δ_pH_) and the electric potential of the IMM (Δ_Ψ_) is used by ATP synthase to synthesize ATP from ADP and P_i_ (Saraste 1999).

Beside their central role in energy production, mitochondria are also engaged in many other important metabolic processes such as β-oxidation of fatty acids (Wanders et al. 2010), steroid synthesis (Miller 2013), generation of Fe-S centers and heme synthesis (Schultz et al. 2010), and metabolic cell signaling (Tait and Green 2012). They are involved in cytosolic Ca^2+^ buffering (Contreras et al. 2010), thermogenesis and apoptosis (Wang and Youle 2009). The mitochondrial electron transport chain is also the major source of reactive oxygen species (ROS) in the cell (Turrens 2003).

Given the complexity of mitochondrial physiology, it is not surprising that mitochondrial dysfunction is associated with the pathogenesis of many serious diseases. Reduction in mitochondrial function characterized by lower mitochondrial membrane potential, changes in ETC complex activities, lowered ATP synthesis, inefficient Ca^2+^ buffering, increased ROS production, altered mitochondrial dynamics or release of pro-apoptotic factors is found during aging and in many chronic diseases (Nicolson 2014; Srivastava 2017), including cardiovascular diseases (Ballinger 2005; Chistiakov et al. 2018), diabetes (Joseph et al. 2012), cancer (Lund et al. 2015) and many metabolic syndromes (Ryter et al. 2014). Because of high energy requirements, neurons are especially vulnerable to mitochondrial dysfunction, as seen in various neurodegenerative disorders such as Alzheimer’s disease (AD), Parkinson’s disease (PD), Huntington’s disease (HD), amyotrophic lateral sclerosis (ALS) and Friedreich’s ataxia (Chaturvedi and Flint Beal 2013; Golpich et al. 2017; Schon and Przedborski 2011).

### Do Mitochondria contain nicotinic acetylcholine receptors?

Nicotine acts as an agonist of nAChRs. nAChRs are pentameric ligand gated-ion channels, that are present on the plasma membrane of neuronal as well as non-excitable cells (Albuquerque et al. 2009). These receptors are expressed in mammals as well as in invertebrates, plants and even bacteria. nAChRs are composed of ten α and four β subunits that can be differently assembled to determine cation selectivity (Gergalova et al. 2012). A study on isolated mitochondria from the liver of C57Bl/6 mice revealed that α7 nAChR subunits are also present in OMM (Gergalova et al. 2014). The same group also reported that mitochondria express α3β2, α4β2, α7β2, α9 nAChR subtypes which engage intra-mitochondrial kinases after the binding of either agonists, competitive antagonists, or positive allosteric modulators (Lykhmus et al. 2014; Lykhmus et al. 2017). As shown by Kalashnyk et al., α7 is the most abundant nAChR subunit in the mitochondrial membrane (Kalashnyk et al. 2012). It is also the most permeable to calcium ions (del Barrio et al. 2011; Shen and Yakel 2009), underlining the relationship between nAChR and Ca^2+^ buffering abilities of mitochondria. It is important to underline that the evidences showing that nAChR can also be localized in the outer mitochondrial membrane (OMM) have been presented only by the group of Skok and co-workers. Taking into account the described methodology for mitochondria isolation from mouse livers, it seems obvious that they isolated crude mitochondrial fractions, containing also mitochondria-associated membranes (like endoplasmic reticulum and/or plasma membranes). Mitochondria isolated in this way were used next to isolate outer and inner mitochondrial membranes. Unfortunately, Gergalova and coworkers did not demonstrate that the OMM preparations were free from plasma membrane contamination (Gergalova et al. 2012). Such a control is crucial to confirm that the nAChR detected in OMM is a mitochondrial protein and does not derive from contamination by plasma membranes. An analysis showing the absence of plasma membrane protein markers such as PMCA, Na^+^/K^+^-ATPase in the OMM preparations would have been helpful in confirming that nAChR is present in the OMM.

Unfortunately, up to now no reports from other laboratories neither confirming nor rebutting Skok’s group findings have been presented. At the moment the studies from other laboratories are needed to confirm such interesting phenomenon.

Previous studies on nAChRs revealed that nicotine can increase the permeability of nAChRs to Ca^2+^, resulting in elevated concentrations of cytosolic Ca^2+^. Furthermore, α7 nAChRs on the outer mitochondrial membrane were shown to regulate voltage-dependent anion channel-mediated Ca^2+^ transport and mitochondrial permeability transition which is responsible for cytochrome c release and the triggering of apoptosis (Wonnacott et al. 2005; Zhang et al. 2004). Nicotine can also act as an intracellular chaperone, favoring the nAChRs’ pentameric assembly and increasing protein glycosylation, which promotes redistribution of nAChR to mitochondria (Sallette et al. 2005). This was reported by Uspenska et al., showing that, in mice consuming nicotine in drinking water for 7 days, the ratio of mitochondrial nAChRs to non-mitochondrial nAChRs in the liver was increased (Uspenska et al. 2017). Nevertheless, additional investigation would still be required to confirm that nicotine effects on mitochondria are actually mediated through nAChR.

## Nicotine and the mitochondrial respiratory chain

Several studies revealed inhibitory effects of nicotine on the OXPHOS machinery. Already in 2001, Cormier et al. demonstrated that nicotine inhibits oxygen consumption of isolated rat brain mitochondria in a concentration-dependent manner (an effect visible already in the concentration range of 1x10^-12^ - 1x10^-9^ M, with the maximal inhibitory effect observed at 1x10^-7^ M nicotine) (Cormier et al. 2001).

This effect of nicotine on mitochondrial oxygen consumption suggested its direct influence on respiratory chain complexes. A [^3^H]-nicotine binding assay revealed that nicotine binds to complex I and inhibits its NADH-ubiquinone reductase activity. Interestingly, the estimated nicotine affinity for mitochondria is higher than its affinity for a cell membrane-bound nAChR. Moreover, the same authors confirmed that the nicotine binding site in complex I is different from the one characterized for rotenone (a known inhibitor of complex I), but is the same as for NADH (Cormier et al. 2001). Nicotine binding results in inhibition of electrons flowing from NADH to complex I (Cormier et al. 2001), which is linked to a detectable decrease in oxygen consumption by mitochondria. Different binding sites for nicotine compared to rotenone mean that competition between nicotine and NADH manifests not only in decreased activity of complex I, but also (in contrast to rotenone) in reduced superoxide generation. Interestingly, the same inhibitory effect of nicotine on mitochondrial respiration has been observed in both, isolated rat brain mitochondria treated directly with nicotine and brain mitochondria isolated from rats that were chronically exposed to nicotine. In both cases, nicotine inhibited ADP-stimulated oxygen consumption (state 3), measured in the presence of glutamate and malate (substrates for complex I). On the other hand, the resting respiration state (state 4), estimated in the presence of glutamate and malate only, was not altered (Cormier et al. 2001). Cormier et al. concluded that the observed inhibitory effect of nicotine on respiratory state 3 was not related to the inhibition of mitochondrial ATP synthase (Cormier et al. 2001).

Most studies investigating the influence of nicotine on mitochondrial metabolism correlated its effect with complex I inhibition. With the use of specific OXPHOS substrates, no alterations of other respiratory chain complexes by nicotine were observed. In contrast to these observations, Das et al. showed that brain mitochondria isolated from Wistar rats treated with nicotine (1 mg/kg/day) for 7 days exhibited decreased activity not only of complex I, but also of complexes II and III of the mitochondrial respiratory chain. Decreased OXPHOS functionality seemed to be caused rather by nicotine modulating enzymatic activity of respiratory complexes than by its effect on the level of OXPHOS complexes. Even a high dose of nicotine (1 mM) had no effect on the levels of individual OXPHOS subunits (Guo et al. 2012).

Direct effects of nicotine on the mitochondrial respiratory chain were also observed in a model of anoxia/reoxygenation. Nicotine added before anoxia/reoxygenation events (5 min of anoxia followed by 5 min of reoxygenation) showed a protective, concentration-dependent effect on the respiratory control ratio (state 3/state 4) in isolated brain mitochondria, increasing the ratio above baseline. Interestingly, when nicotine was added after anoxia but just before reoxygenation, it was not able to preserve mitochondrial functions (Cormier et al. 2001). Two years later, the same group showed that nicotine administration in rats for 7 days also provided protection against anoxia/reoxygenation events (Cormier et al. 2003).

The effects of nicotine and smoking on whole body metabolism (e.g. increased lipid metabolism) was first documented almost three decades ago (Jacob et al. 1988; Jensen et al. 1995) as well as more recently (Chiolero et al. 2008). The direct influence of nicotine on hepatic metabolic function was extensively studied by Dewar et al. at different levels of complexity (Dewar et al. 2002). They were first to show that the effect of nicotine on oxygen consumption depends on fed or fasting state of the animals. Infusion of nicotine (at concentrations ranging from 85 μM up to 850 μM) increased oxygen uptake/consumption in perfused livers of fed rats only. This increased oxygen consumption was accompanied by decreased lactate and pyruvate production, suggesting a decrease in the glycolysis rate. Interestingly, there was practically no effect of nicotine on the basal rate of oxygen consumption in perfused livers from rats fasted for 24 h. Inhibition of the mitochondrial respiratory chain by potassium cyanide confirmed that the changes in oxygen uptake observed in perfused livers from fed animals were of mitochondrial origin. Moreover, the effect of nicotine was abolished by infusion of atractyloside, an inhibitor of adenine nucleotide translocase, suggesting that the nicotine induced-increase of oxygen consumption depends on ADP supply to mitochondria and is related to the changes in mitochondrial respiration state 3. Similar to the phenomenon observed for perfused livers, 1 mM nicotine increased oxygen consumption by 30% in hepatocytes isolated from livers of fed rats. In perfused livers, nicotine had an effect on oxygen consumption of hepatocytes isolated from livers of fasted rats. A surprising result obtained by Dewar et al., which is in contrast to other reports, concerns the effect of nicotine on isolated mitochondria. They claim that there is no effect of nicotine (at concentrations ranging from 50 μM up to 1.25 mM) on oxygen consumption of isolated rat liver mitochondria. The difference can be explained by the fact that Dewar and colleagues measured mitochondrial respiration only in the presence of succinate as OXPHOS substrate. The omission of glutamate and malate as substrates in this study made an evaluation of the effect of nicotine on complex I of the respiratory chain impossible. Taking into account that practically 80% nicotine is metabolized in the liver to its metabolite cotinine and that cotinine concentrations *in vivo* are nearly 10 times higher than nicotine, Dewar et al. studied also the effect of 1.25 mM cotinine on mitochondrial oxygen consumption. They found that cotinine had a similar effect on hepatic oxygen uptake; however, its effect on glycolysis was different from the one observed with nicotine (Dewar et al. 2002). This observation is in contrast with the data of Cormier et al. who reported that cotinine had no effect on either state 3 or state 4 in isolated rat brain mitochondria (Cormier et al. 2001). This difference in the effect of cotinine may be due to the fact that the studies used mitochondria isolated from liver or brain, respectively (Cormier et al. 2001; Dewar et al. 2002).

A substantial number of reports describing the effect of nicotine on mitochondria is limited to the use of mitochondria isolated from whole brains. A more detailed study was performed by Wang et al., investigating the changes in gene expression patterns in different brain regions (amygdala, hippocampus, nucleus accumbens, prefrontal cortex, striatum, and ventral tegmental area) from female Sprague-Dawley rats exposed to nicotine (3.15 mg/kg/day) for 7 days (Wang et al. 2009). This study revealed that chronic nicotine administration modulates the expression of several genes encoding individual subunits of the mitochondrial respiratory chain (Table 1).

**Table 1:** Summary of the genes differentially expressed in different brain areas of rats chronically exposed to nicotine (Wang et al. 2009).

	**OXPHOS genes**		
**Brain region**	**Up-regulated**	**Down-regulated**	**Not changed**
Amygdala	*Mt-nd1, Mt-nd4, Mt-nd6, Mt-cyb, Mt-co1, Mt-co2, Mt-co3, Atp5j,*		*Mt-nd2, Atp5b*
Hippocampus	*Mt-nd1, Mt-nd2, Mt-nd4, Mt-nd6, Mt-co1, Mt-co2, Mt-co3, Atp5j,*		*Mt-cyb, Atp5b*
Nucleus accumbens		*Mt-nd1, Mt-nd2, Mt-nd4, Mt-nd6, Mt-cyb, Mt-co1, Mt-co2, Mt-co3, Atp5b, Atp5j,*	
Prefrontal cortex	*Mt-nd1, Mt-nd2, Mt-nd4, Mt-nd6, Mt-cyb, Mt-co1, Mt-co2, Mt-co3, Atp5b, Atp5j,*		
Striatum		*Mt-nd1, Mt-nd2, Mt-nd4, Mt-nd6, Mt-co1, Atp5j*	*Mt-cyb, Mt-co3, Atp5b*
Ventral tegmental area	*Mt-nd1, Mt-nd2, Mt-nd4, Mt-nd6, Mt-cyb, Mt-co1, Mt-co3, Atp5b, Atp5j*		

Abbreviations: **Complex I**: *Mt-nd1,* (NADH dehydrogenase subunit I); *Mt-nd2*, (NADH dehydrogenase subunit II); *Mt-nd4*, (NADH dehydrogenase subunit IV); *Mt-nd6, (NADH dehydrogenase subunit VI);***Complex III**: *Mt-cyb*, (cytochrome b); **Complex IV**: *Mt-co1*, (cytochrome c oxidase subunit I); *Mt-co2*, (cytochrome c oxidase subunit II); *Mt-co3* (cytochrome c oxidase subunit III); **ATP synthase**: *Atp5b*, (ATP synthase, F1 complex, β subunit); *Atp5j*, (ATP synthase, F0 complex, subunit F6)

By demonstrating the differential impact of nicotine exposure on OXPHOS gene expression profiles in different brain regions, Wang et al. highlighted the limitation of previously reported nicotine effects using only mitochondria isolated from whole brain tissue (Wang et al. 2009). This aspect should be taken into consideration in future studies. Moreover, such differences in the response of individual brain regions to nicotine treatment should be also represented as various alterations in mitochondrial bioenergetics metabolism and different modulation of OXPHOS complexes activities.

As was already mentioned, not only complex I can be affected by nicotine. Similar to Das et al. showing nicotine-induced inhibition of complexes II and III (Das et al. 2009), recent studies of Raval et al. also identified complex IV as a possible target for nicotine (Raval et al. 2012). They found that mitochondria isolated from hippocampi of rats exposed to nicotine (4.5 mg/kg/day) for 16 days had a reduced rate of mitochondrial respiration (state 3 in presence of ADP) in the presence of pyruvate/malate (substrates for complex I) and ascorbate/N,N,N’,N’-tetramethyl-p-phenylenediamine (TMPD) (substrates for complex IV). These results together with the other reports suggest that nicotine induces inhibition of complexes I and IV. However, spectrophotometric measurement of the activities of individual respiratory chain complexes revealed that nicotine had no effect on the activity of complexes I, II and III. Interestingly, Raval et al. observed also a decreased activity of complex IV in hippocampal mitochondria isolated from rats chronically treated with nicotine. There were no differences in the levels of representative subunits of OXPHOS in mitochondria from nicotine-treated rats. This means that observed changes in the activities are not related to the level of individual complexes. Moreover, decreased oxygen consumption in the presence of pyruvate/malate without alterations in spectrophotometrically measured complex I activity suggests that the observed inhibition of mitochondrial respiration with complex I substrates could be related to complex IV inhibition. Complex IV, being the last step in electron transport from NADH to oxygen, could be – when inhibited – the limiting step in the respiratory chain regardless of OXPHOS substrates used in the assay (Raval et al. 2012). Recently, Lei et al. also proposed an involvement of complex III in nicotine-mediated inhibitory effect on OXPHOS (Lei et al. 2017).

From these data, it appears that nicotine affects the mitochondrial respiratory chain beyond complex I inhibition. While for complex I the character of nicotine interaction was determined, the mechanisms leading to the observed inhibitory effects of other complexes still required further investigation. Apart from a direct interaction with respiratory complexes, prolonged nicotine exposure could imply changes of respiratory chain subunit levels, indicative of rearrangements within the OXPHOS machinery. This is likely a cellular response aimed at adapting to decreased mitochondrial respiratory chain efficiency. Changes in OXPHOS function usually have broader repercussions for other aspects of mitochondrial function such as the ATP synthesis and ROS production. Nicotine impact of ROS production is reviewed in section 2.

### Nicotine – an uncoupling effect on mitochondrial respiration

Taking into account the inhibitory effect of nicotine on OXPHOS, a report describing an uncoupling effect of nicotine observed in brown adipose tissue (BAT) mitochondria is particularly interesting. Two-week nicotine treatment of MSG-obese mice (neonatally treated with monosodium-L-glutamate, these mice are known to become obese without hyperphagia) caused increased guanosine-5'-diphosphate (GDP) binding to mitochondria and increased oxygen consumption of BAT mitochondria. The authors claim that nicotine seems to uncouple mitochondrial respiration via uncoupling protein 1 (UCP1) which consequently up-regulated BAT thermogenesis and significantly reduced bodyweight of MSG obese mice without affecting food intake (Yoshida et al. 1990).

### Protective effect of nicotine against neurodegenerative disorders mediated by OXPHOS inhibition

The effect of nicotine on the mitochondrial respiratory chain has been observed in certain neurodegenerative diseases such as PD, which is a long-term neurodegenerative disorder related to the death of dopaminergic neurons in the substantia nigra, as well as in the case of AD, where, however, such an association is still controversial. Increased neuronal death in PD patients harboring *PARK2* mutations is correlated with perturbations in an ability to dispose of dysfunctional mitochondria producing extensive amounts of ROS. Interestingly, in PD patients reduced activity of mitochondrial respiratory chain complex I and practically lower activity of all other OXPHOS complexes in brains of AD patients have been observed (Parker et al. 1994).

The beneficial effect (i.e., an alleviation of symptoms of neurodegenerative disorders) of nicotine in smokers can be discussed at different levels: a) nicotine can be considered as an antioxidant (Linert et al. 1999), which is able to block Fenton’s reaction mostly by Fe chelation, or acts as a scavenger of H_2_O_2_ (the direct interplay between nicotine and ROS will be discussed in another section of this review); b) nicotine could have a direct effect on OXPHOS complexes and, in this way, modulate mitochondrial bioenergetics and/or influence ROS production by respiratory chain complexes. The second option seems to be highly probable, because mitochondria are a target for nicotine, especially in brain, lung and liver as previously shown (Cormier et al. 2001; Jung et al. 1999; Xie et al. 2005). Independent of the effect on OXPHOS, neuroprotective effects of nicotine in rat and mouse models of PD were shown to be related to the induction of neurotrophic factors, which in turn result in the recovery of dopaminergic neuron loss (Maggio et al. 1998; Maggio et al. 1997). Moreover, nicotine may elicit a neuroprotective effect in PD models by inhibiting PARP-1 and caspase-3 cleavage (Lu et al. 2017). Furthermore, in the rotenone model of PD, co-treatment of rats with rotenone and nicotine for 7 or 14 days resulted in limited deleterious effects of rotenone at the level of mitochondrial parameters. There was a significant increase in the respiratory ratio of isolated brain mitochondria in the nicotine-treated animals (Cormier et al. 2001; Cormier et al. 2003). This increase was, however, still lower than that observed in mitochondria isolated from the brains of control animals. This inhibitory, ROS-free effect of nicotine on complex I may, at least in part, explain the beneficial effect of nicotine observed in smokers suffering from neurodegenerative diseases.

## Nicotine and oxidative stress

Intracellular ROS formation is a natural and metabolic process, occurring in different intracellular compartments, including mitochondria (Holmström and Finkel 2014). ROS may act as signaling molecules, since the extent of their production reflects metabolic activity of the pathways in which they are formed. However, excess accumulation of ROS may lead to oxidative stress which can be induced by a broad range of agents including nicotine.

The mitochondrial electron transport chain is an important intracellular source of ROS (Munro and Treberg 2017). It contains several sites, where electrons can escape the routine sequence of reactions through a series of redox centers and react with oxygen, leading to formation of superoxide radical ions (O_2_^.-^ ). O_2_^.-^ can give rise to other ROS. Its detoxification includes dismutation to H_2_O_2_ by superoxide dismutases (SOD) followed by catalase-mediated decomposition of H_2_O_2_.

Nicotine was reported to modulate intracellular ROS production in both directions: induction and attenuation, depending on ROS source and physiological state of the cell. Since nicotine can bind and inhibit respiratory chain complex I, it can potentially affect respiratory chain-mediated ROS production. Experiments with isolated rat brain mitochondria and submitochondrial particles showed that complex I inhibition by nicotine is accompanied by lower electron leakage from complex I and thus, lower ROS production driven by complex I substrates (Cormier et al. 2001; Xie et al. 2005). It is also worth noting, that nicotine can complex with free Fe, reducing its reactivity (Bridge et al. 2004). Fenton’s reaction involving Fe^2+^ and H_2_O_2_ is responsible for the conversion of H_2_O_2_ to very reactive hydroxyl radicals in mitochondria (Munro and Treberg 2017). Nicotine could also potentially affect this aspect of mitochondrial ROS metabolism; however, this issue has not been addressed so far.

Isolated mitochondria facilitate the analysis of mechanisms underlying interactions between substances of interest and mitochondrial enzymes. However, this experimental model usually operates within physiological extremes, with either uncoupled or highly polarized mitochondria. Thus, the obtained results should always be interpreted in parallel with supplementary observations made in intact cells and/or using animal models.

Numerous studies on cultured cells showed that nicotine treatment increases cytosolic ROS levels and leads to oxidative stress. This was observed in a variety of cell lines, including astrocytes (Ande et al. 2012), renal proximal tubule cells (Arany et al. 2011; Arany et al. 2016; Hall et al. 2017), mesangial cells (Jaimes et al. 2007), podocytes (Lan et al. 2016), peritoneal macrophages (Mahapatra et al. 2009), bronchial and lung epithelial cells (Bodas et al. 2016; Zanetti et al. 2014), lung carcinoma (Guo et al. 2012) and colon adenocarcinoma cell lines (Crowley-Weber et al. 2003; Pelissier-Rota et al. 2015). In most studies using cell lines, nicotine-mediated induction of intracellular ROS was detectable at nicotine concentrations around 1 μM. In studies with renal tubule cells, 200 μM nicotine was sufficient (Arany et al. 2016), while millimolar nicotine concentrations were used in human BEAS-2B bronchial epithelial cells and mouse peritoneal macrophages to stimulate ROS production (Bodas et al. 2016; Mahapatra et al. 2009). The extent of increase in ROS levels by nicotine is also time-dependent. In human SVG-A fetal astrocytes the maximal increase in ROS levels was detected after 30 minutes of nicotine treatment, while it was less pronounced during longer incubations, possibly due to increased cytosolic SOD (SOD1) expression – a potential adaptive mechanism (Ande et al. 2012). Effects of nicotine treatment on antioxidant enzymes were demonstrated also in HepG2 cells, where a 24-h treatment decreased SOD and glutathione reductase (GR) activities, but increased activities of catalase (CAT) and glutathione peroxidase (GPx) (Yarahmadi et al. 2017).

It has been shown that nicotine-induced increases in intracellular ROS levels are involved in redox-sensitive signaling. In TKPTS renal proximal tubule cells, nicotine treatment led to the activation of transcription factor AP-1 in a JNK-dependent manner (Arany et al. 2011). In A549 lung carcinoma cells, ROS level elevation caused by nicotine treatment resulted in the phosphorylation of Akt and ERK kinases and to the stabilization of hypoxia-inducible factor 1α (HIF-1α) (Guo et al. 2012), whereas in HepG2 hepatocytes, nicotine treatment was shown to activate the NF-κB response element (Crowley-Weber et al. 2003). Increased expression of the NF-κB p65 subunit upon nicotine treatment was reported in HK-2 renal proximal tubule cells and was dependent on increased ROS levels and subsequent phosphorylation of ERK and JNK kinases (Kim et al. 2016).

Nicotine-mediated increases in intracellular ROS levels were attributed to triggering apoptosis (Kim et al. 2016; Lan et al. 2016; Pelissier-Rota et al. 2015; Zanetti et al. 2014), inducing endoplasmic reticulum (ER) stress (Pelissier-Rota et al. 2015), and impairing (Bodas et al. 2016) as well as stimulating autophagy (Pelissier-Rota et al. 2015).

Despite the large number of studies reporting increased intracellular ROS levels upon nicotine treatment, only few tried to determine which specific intracellular ROS source was responsible for this increase. The results are not consistent between studies, and it seems that nicotine can influence various intracellular ROS sources to different extent in different cell types (Table 2). In SVG-A astrocytes, the nicotine-mediated increase in intracellular ROS levels was partially prevented by the inhibition of cytochrome P450 2A6 enzyme with tryptamine (Ande et al. 2012), while in podocytes, it was abolished by the NADPH oxidase inhibitor VAS2870 (Lan et al. 2016). In MLA-12 lung epithelial cells and primary alveolar epithelial cells, nicotine-induced ROS production was also sensitive to NADPH oxidase inhibition, silencing or knock-out (Zanetti et al. 2014). In TKPTS mouse renal proximal tubule cells challenged with H_2_O_2_, nicotine caused further increases in ROS levels, which were not affected by the xanthine oxidase inhibitor allopurinol, but were sensitive to antimycin A (an inhibitor of mitochondrial respiratory chain complex III) and diphenyleneiodonium (DPI) (Arany et al. 2011). Although DPI was used in this study as NADPH oxidase inhibitor, this chemical appears to have also other activities, including decreasing superoxide synthesis by respiratory chain complex I (Lambert et al. 2008). Thus, the interpretation of this result is not unequivocal. An increase in mitochondrial ROS level was measured in nicotine-treated HT29 adenocarcinoma cells, and this increase was abolished by rotenone and myxothiazol (complex I and complex III inhibitors, respectively), indicating that ROS originated from the respiratory chain (Pelissier-Rota et al. 2015). This study also showed that the mitochondrially targeted antioxidant MitoTEMPO prevented nicotine-induced ER stress, autophagy and apoptosis, pointing to mitochondrial ROS as triggers of these nicotine effects. In human A549 lung carcinoma cells, mitochondrial ROS were shown to mediate nicotine-induced activation of the transcription factor HIF-1, which could be abolished by the antioxidant N-acetylcysteine, the respiratory chain inhibitor rotenone or overexpression of mitochondrially targeted CAT. In contrast, overexpression of mitochondrial superoxide dismutase (SOD2), which increases intramitochondrial H_2_O_2_ levels, enhanced HIF-1α stabilization and HIF-1 activation following nicotine treatment (Guo et al. 2012). In nicotine-treated NRK52E renal proximal tubule cells, increases in ROS levels and cell injury were abolished by stimulation of SOD2 expression, pointing to mitochondrial ROS as mediators of cell injury caused by nicotine (Hall et al. 2017).

**Table 2:** Identification of ROS sources responsible for nicotine-induced intracellular ROS increases

**Cell line**	**Nicotine dose**	**Treatment duration**	**Postulated ROS source**	**Nicotine effect**	**Verification of ROS source**	**Ref.**
SVG-A astrocytes, human	1 μM	30 min	cyt P450	cytosolic ROS increase	prevented by cyt P450 inhibitor, tryptamine	(Ande et al. 2012)
Podocytes, human	0.1-10 μM	12 h	NOX	cytosolic ROS increase	prevented by NOX inhibitor, VAS2870	(Lan et al. 2016)
MLA-12 lung epithelial cells, mouse	1 μM 10 μM 100 μM	48 h	NOX; secondary effect on mitochondrial ROS	cytosolic and mitochondrial superoxide increase	prevented by NOX inhibitor, GKT136901 or NOX silencing	(Zanetti et al. 2014)
Primary alveolar epithelial cells, human	100 nM	48 h	NOX	cytosolic superoxide increase	not present in Nox^-/-^ cells	
HT 29 adeno-carcinoma, human	100 nM	5 and 30 min	mitochondria; increased ROS production probably due to stimulation of calcium fluxes	mitochondrial ROS increase	diminished by rotenone and myxothiazol, prevented by MitoTEMPO and EGTA	(Pelissier-Rota et al. 2015)
A549 lung carcinoma, human	1 μM	3 h	mitochondria	HIF-1 alpha activation	prevented by N-acetylcysteine, rotenone and overexpression of mitochondrially-targeted catalase; enhanced by overexpression of mitochondrial SOD	(Guo et al. 2012)

Abbreviations: ROS, reactive oxygen species; NOX, NADPH oxidase; cyt P450, cytochrome P450; EGTA, ethylene glycol-bis(β-aminoethyl ether)-N,N,N′,N′-tetraacetic acid; SOD, superoxide dismutase; Ref., references.

Another aspect of how nicotine affects mitochondrial ROS production is its effect on p66Shc, which was extensively studied by Arany et al. (Arany et al. 2013; Arany et al. 2016). p66Shc is an adaptor protein and a negative regulator of proliferation. Apart from that function, upon Ser36 phosphorylation, p66Shc translocates to mitochondria, where it stimulates ROS production, probably via interaction with cytochrome c (Migliaccio et al. 1999). This action of p66Shc was often attributed to triggering a vicious cycle of ROS-induced increase in ROS levels leading to an aggravation of oxidative injury under various stress conditions (Migliaccio et al. 1999). In renal proximal tubule cells, nicotine increased p66Shc promoter activity in a p53- and DNA hypomethylation-dependent manner (Arany et al. 2013). Other results by this group suggest that stimulation of p66Shc expression in kidney cells underlies nicotine-mediated aggravation of oxidative stress caused by other toxic insults such as ischemia-reperfusion, H_2_O_2_ or oleic acid treatment (Arany et al. 2013; Arany et al. 2016).

In general, the studies with cultured cells demonstrate that nicotine treatment increases, rather than decreases cytosolic and mitochondrial ROS levels, which is contrary to what was shown on isolated mitochondria. However, there are experimental systems, where nicotine appeared to diminish ROS levels caused by other toxic insults, as is the case of β-amyloid-treated primary hippocampal cell cultures (Liu and Zhao 2004). It has to be mentioned here that the nicotine effects observed in cell cultures do not reflect exclusively a direct interaction of nicotine with mitochondrial enzymes, but can also result from the activation of signaling pathways secondary to mitochondrial function or ROS production, which are not observable in experiments with isolated mitochondria.

In cell culture systems, nicotine mostly increased ROS levels and mitochondria were one of the potential sources of these ROS, besides NADPH oxidases and cytochrome P450 enzymes. Nicotine-induced ROS were shown to activate various intracellular signaling pathways, which participate in adaptation to oxidative stress, but can also lead to cell damage and apoptosis.

Oxidative stress induced by nicotine was also investigated in animal models. Oxidative injury as well as alterations in the cellular antioxidant defense system were shown in various tissues of nicotine-treated animals. The most examined markers of oxidative stress are ROS production (Barros et al. 2007; Hritcu et al. 2009; Toledano et al. 2014), activities of antioxidant defense system components (Das et al. 2009; Hritcu et al. 2009; Toledano et al. 2014), lipid peroxidation (Barros et al. 2007; Das et al. 2009; Hritcu et al. 2009; Toledano et al. 2014), protein carbonylation (Das et al. 2009) and DNA damage (Barros et al. 2007; Hritcu et al. 2009).

Taking into account that mitochondria are the organelles affected by nicotine treatment, as well as the high metabolic activity of brain, it seems that this organ may be particularly susceptible to oxidative stress induced by nicotine. The available literature corroborates this with data showing different sensitivities of specific brain regions to nicotine-induced oxidative stress *in vivo*. Hritcu et al. demonstrated augmented ROS production after nicotine treatment (0.3 mg/kg/day for 7 days) in rat temporal cortex resulting in negative effects on short- and long-term memories (Hritcu et al. 2009). However, a study by Barros et al. indicated no nicotine effect (0.3 mg/kg or 1 mg/kg/day) on ROS concentration in rat cortex, while ROS levels were increased in the hippocampus of rats exposed to the higher nicotine doses for 9 days (Barros et al. 2007). Furthermore, this study showed that higher doses of nicotine facilitated short- and long-term memory, but also exerted DNA damage and lipid peroxidation. Another study demonstrated that nicotine (1 mg/kg/day for 7 days) caused significant augmentation of lipid peroxidation and protein carbonylation in different regions of rat brain such as the cerebellum, cerebral hemispheres and diencephalons (Das et al. 2009). After supplementation with antioxidants, the extent of those impairments was decreased. Data by Toledano et al. suggest that changes in the level of specific SOD isoforms in response to nicotine treatment depend on brain region and hence the different cell types present in those regions (Toledano et al. 2014). Nevertheless, the levels and activities of antioxidant enzymes such as SOD, CAT, GR, GPx and glutathione S-transferase (GST) were reportedly decreased following nicotine treatment (Das et al. 2009; Hritcu et al. 2009).

Hallmarks of oxidative stress and damage were shown also in other organs of animals receiving nicotine treatment. Increased levels of thiobarbituric acid reactive substances (TBARS), hydroperoxides and nitric oxide, decreased GSH levels as well as lower activities of antioxidant enzymes (SOD, CAT and GPx) were detected in plasma, lungs, liver and kidney of rats after 22 weeks of subcutaneous nicotine administration (Muthukumaran et al. 2008). Additionally, an increase in the oxidative damage markers malondialdehyde (MDA) and nitrotyrosine was detected in mouse kidneys following nicotine administration in drinking water for 4 weeks (Arany et al. 2011). However, in another study, performed in rats, subcutaneous injection of nicotine for 6.5 weeks had no effect on kidney MDA levels, whereas it increased levels of this lipid peroxidation marker in liver (Husain et al. 2001). At the same time, distinct effects on the antioxidant systems in these two organs were noted: Nicotine treatment increased SOD and decreased CAT activity in the liver, while in kidney, the effects were the opposite (Husain et al. 2001). Interestingly, a study performed in mice, applying a similar treatment duration (5 weeks), but with a much higher nicotine dose administered intraperitoneally, demonstrated a decrease in SOD, CAT and GPx activities in both organs, accompanied by GSH depletion and elevated MDA levels (Kim et al. 2017). The discrepancies in these results can be caused by differences in the route of administration, nicotine dose and treatment duration. However, it is clear that nicotine induces oxidative stress and affects antioxidant defense systems in different tissues. It is also worth noting, that animals of distinct age groups may exhibit different toxic manifestation of nicotine-induced oxidative stress (Jain and Flora 2011).

Lungs are of particular interest regarding the effect of nicotine. It has been shown that long-term nicotine administration in rats (2.5 mg/kg/day for 18 weeks) caused histopathological changes as well as oxidative damage in the lungs (Dhouib et al. 2015). Specifically, nicotine treatment significantly increased lipid peroxidation as well as SOD and CAT activities in pulmonary tissue (Dhouib et al. 2015). Other studies applying lower doses of nicotine (0.5 mg/kg/day) for 3 or 4 weeks reported increased lipid peroxidation, decreased GSH levels and decreased SOD and GR activities in lungs and liver (El-Sokkary et al. 2007; Erat et al. 2007). Nicotine effects were abolished by co-treatment with antioxidants such as vitamin E (Erat et al. 2007) or melatonin (El-Sokkary et al. 2007).

Together, these data indicate that nicotine causes oxidative stress and thereby mediates oxidative damage of cellular macromolecules. The overall effects of nicotine, however, depend on many factors such as type of organ, region of the brain or even age of examined animals. Nevertheless, it is worth noting that, in the context of neurological disorders such as PD and AD, nicotine may have a dual role – either as a neurotoxic or neuroprotective factor (Linert et al. 1999; Quik 2004). The beneficial effect of nicotine is prominent mostly in relation to motor and cognitive skills as well as the improvement of information processing and attention processes and could possibly be explained by synergistic action of nicotine with antioxidants (Linert et al. 1999; Sack et al. 1996).

The exact mechanism(s) and potential involvement of mitochondria in the development of nicotine-induced oxidative stress observed in *in vivo* studies need deeper investigations. However, studies in cell cultures point to mitochondria as one of the possible mediators of intracellular ROS increase observed upon nicotine exposure. Nicotine-induced ROS were also shown to participate in intracellular signaling, related mostly to stress responses, which is particularly interesting also in the context of the beneficial nicotine effects observed in some experimental models. ROS are important mediators of adaptive responses allowing for cell survival under stress conditions. However, the role of ROS in nicotine mediated cytoprotection has not been addressed so far.

## Nicotine and calcium buffering

Calcium-based signalling is a versatile mechanism through which extra- and intracellular messengers modify various biological processes. In the regulation of calcium concentration, a specific machinery including exchangers, ion channels and pumps is involved and mediates Ca^2+^ fluxes across the plasma membrane. Additionally, two organelles take part in intracellular calcium handling: the ER and mitochondria. The ER acts as a major intracellular Ca^2+^ store, whereas mitochondria, due to their ability of intensive calcium uptake, can buffer excessive ions from the cytosol and transfer it to the ER through mitochondria-ER contact sites. The ability of mitochondria to capture calcium is involved in energy production, shaping cytosolic calcium rises, ROS generation and triggering apoptosis (Contreras et al. 2010; Santo-Domingo and Demaurex 2010; Wang et al. 2009). These mechanisms are essential for proper cell function and are widely recognized as fundamental for cell signaling involved in aging, cell proliferation and cell death. Moreover, emerging evidence indicates causation in impaired calcium homeostasis and neurodegenerative diseases such as AD, PD or ALS. The increasing knowledge about the mechanisms underlying the degenerative processes linked to those diseases has triggered the search for new compounds capable of halting or decreasing neural deterioration. Nicotine has been proposed as one of these neuroprotective compounds (Godoy et al. 2018).

Nicotine exerts its biological function mainly through stimulation of nAChR located in the cell membrane, which leads to an increase in intracellular Ca^2+^ levels. This change in cytosolic calcium levels may influence Ca^2+^ flux into mitochondria, which subsequently leads to changes in mitochondrial metabolism (Guo et al. 2012). The homeostasis of Ca^2+^ is fundamental for proper mitochondrial function and signaling pathways critical to cell life. It has been recently demonstrated that different nAChR subtypes are present in OMM and regulate Ca^2+^ accumulation and cytochrome c release stimulated with a high calcium dose (Gergalova et al. 2012).

Mitochondrial calcium buffering is important for decreasing cytosolic levels of these ions and is involved in the regulation of many Ca^2+^-dependent processes. A study in isolated mouse mitochondria showed that the mitochondrial ability to buffer calcium was reduced by 20% following treatment with the nAChR agonists choline, acetylcholine and PNU282987 (Gergalova et al. 2012). In turn, Barrio et al., using bovine chromaffin cells expressing mitochondrialy or cytosolic-targeted aequorin, showed that activation of nAChR by nicotine evoked larger changes in mitochondrial than in cytosolic calcium levels (del Barrio et al. 2011). This implies that differential activation of nAChRs has distinct effects on mitochondrial calcium handling. Mitochondrial calcium buffering occurs through the mitochondrial calcium uniporter (Santo-Domingo and Demaurex 2010) and additionally, by the mitochondrial outer membrane voltage-dependent anion channel, VDAC, that facilitates entry of Ca^2+^ to mitochondrial intermembrane space. VDAC was postulated to interact with α7-nAChR (the isoform considered to be the most abundant in the mitochondrial membrane), which implies the involvement of α7-nAChR in mitochondria-driven apoptosis due to high calcium exposure (Gergalova et al. 2012).

Mitochondrial calcium overload provokes mitochondrial swelling, rupture of the OMM and release of intermembrane components such as cytochrome c (Chernyavsky et al. 2015b). Since nAChRs participate in the regulation of mitochondrial calcium buffering and can interact with VDAC, it is likely that nicotine and other nAChR agonists modulate apoptotic processes. Lykhmus et al. showed that activation of α7-nAChR by different agonists affects PI_3_K/Akt kinases and decreases cytochrome c release upon mitochondrial permeability transition pore (mPTP) activation following calcium stimulation (Lykhmus et al. 2014). Other studies reported anti-apoptotic properties of mitochondrial nAChRs. For example, freshly isolated mitochondria from nicotine-consuming mice were shown to be less susceptible to Ca^2+^-driven cytochrome c release (Uspenska et al. 2017). Chernyavsky et al. showed that nicotine can permeate lung cells and activate mitochondrial nAChR, resulting in inhibition of mPTP opening and abolishing cytochrome c release (Chernyavsky et al. 2015b). This observation was confirmed using other nAChR agonists, showing that receptor activation at the mitochondrial membrane activates signaling pathways similar to receptor activation at the cell membrane, resulting in inhibition of calcium-induced, mitochondria-driven apoptosis (Gergalova et al. 2014). On the other hand, Xie et al. showed that mitochondrial swelling after calcium stimulation in SH-SY5Y cells was inhibited by nicotine in an nAChR-independent manner (the cells were pre-treated with mecamylamine, a non-selective nAChR inhibitor) (Xie et al. 2005).

Nicotine can directly or indirectly trigger various intracellular signaling pathways, including the PKC, MAPK, PI_3_K/Akt, CaMKII pathways among others. Moreover, nicotine has an impact on the variation of cytosolic calcium concentration, which may influence mitochondrial calcium homeostasis. Ca^2+^ homeostasis is fundamental to the function of mitochondria and is related to the generation of ATP, ROS and mitochondrial dynamics (Lykhmus et al. 2014; Wang et al. 2009). Godoy et al. showed that nicotine stimulation affected the association of mitochondria with the ER, increasing the co-localization of mitochondria with inositol (1,4,5)-triphosphate receptor (IP_3_R, a Ca^2+^-release channel from the ER) (Godoy et al. 2018). It seems that modulation of mitochondrial dynamics by nicotine regulates Ca^2+^ efflux from the mitochondria through the Na^+/^Ca^2+^ exchanger to continuously fill the ER. Thus, repeated nAChR activation can enhance calcium release from mitochondria (Shen and Yakel 2009).

Mitochondrial calcium buffering is a key feature of cell life. It has important implications in many cellular processes and determines cell health. The available experimental evidence shows that nicotine influences intracellular calcium fluxes and calcium-dependent signaling due to stimulation of nAChR. This has also consequences for mitochondrial calcium buffering.

## Nicotine and mitochondrial signalling

### Mitochondrial dynamics, biogenesis, quality control

Mitochondrial networks form a dynamic structure, which constantly undergoes opposite processes of fusion and fission. The proper balance between mitochondrial fusion and fission is very important for maintaining cell homeostasis (Roy et al. 2015). Mitochondrial fusion ensures the inheritance and maintenance of mtDNA as well as gene products among the mitochondrial network by mixing content of fused mitochondria to maintain a homogenous population of these organelles in the cell. Mitochondrial fission is involved in cell division to provide equal distribution of mitochondria to daughter cells, facilitates mitochondrial transport along the cytoskeleton by producing smaller organelles, allows for the release of cytochrome c from mitochondria after apoptosis induction and plays a role in mitochondrial quality control by removal of the non-functional fragments of the mitochondrial network (Westermann 2010; Youle and van der Bliek 2012). Since damaged mitochondria in the cell are removed during mitophagy, they are replaced by growth of already existing organelles in the process of biogenesis (Stotland and Gottlieb 2015).

Disturbances in mitochondrial fusion, fission as well as in mitophagy are linked to the pathophysiology of neurodegenerative diseases such as AD, PD and ALS (Aufschnaiter et al. 2017). The influence of nicotine on mitochondrial dynamics was investigated using cultured hippocampal neurons (Godoy et al. 2018) as well as NT2/D1 pluripotent human testicular embryonal carcinoma cells (Hirata et al. 2016). In hippocampal neurons, nicotine at concentrations of 2.5 and 10 μM caused dynamic, time-dependent changes in mitochondrial morphology: Prior to treatment, 25% of the cultured neurons displayed a subpopulation of small mitochondria. After 30 min of nicotine exposure, the fraction of neurons containing small mitochondria increased to 70%. This effect was transient, because after 120 min of nicotine exposure, the proportion of neurons with small mitochondria was similar to that seen prior nicotine treatment. In these conditions, nicotine did not induce changes in the mitochondrial membrane potential. These experiments also revealed that changes in mitochondrial morphology following nicotine treatment involved Drp1, which plays a main role in mitochondrial fission. In addition, nicotine treatment (at concentrations ranging from 2.5 to 25 μM) for 2 h resulted in a dose-dependent increase in Drp1 phosphorylation at Ser616. Elevated levels of p-Drp1 following nicotine treatment may be a consequence of increased cytosolic Ca^2+^, resulting in activation of calmodulin-dependent protein kinase II (CaMKII), which is involved in Drp1 phosphorylation at Ser616. The level of Fis1, another protein involved in mitochondrial fission, was increased, albeit not significantly, following nicotine exposure (Godoy et al. 2018).

In cells, mitochondria are transported along cytoskeletal tracks by interaction with acetylated microtubules (MTs). Upon nicotine treatment (5 μM and 10 μM for 1 h), the co-localization of mitochondria with MTs decreased, indicative of the dissociation of mitochondria from MTs. The level of mitofusin 2 (Mfn 2), a protein involved in mitochondrial dynamics responsible for mitochondrial fusion and tethering of mitochondria to ER, was also not significantly changed following nicotine treatment (Godoy et al. 2018). On the other hand, treatment of NT2/D1 cells with 10 μM nicotine for 24 h resulted in decreased levels of Mfn1 and Mfn2, which in turn increased mitochondrial fission. The levels of Fis1, Drp1 and Opa1 (protein involved in mitochondrial fusion, residing in IMM) did not change upon nicotine treatment, suggesting that mitochondrial fission was upregulated due to Mfn1 and Mfn2 degradation. Treatment with a nonselective nAChR antagonist inhibited this nicotine effect, underlining its nAChR-dependent manner (Hirata et al. 2016).

Nicotine acting via a nAChR-dependent mechanism was proposed to cause dysfunction in mitochondrial quality control, since mitofusin degradation occurs during mitophagy, a process induced by decreased Δ_Ψ_ (Hirata et al. 2016). This reduction in Δ_Ψ_ may lead to the translocation of Parkin to mitochondria, where it ubiquitinates Mfn1 and Mfn2, thereby targeting these proteins for proteasomal degradation and finally leading to removal of damaged mitochondria during mitophagy (Ni et al. 2015; Stotland and Gottlieb 2015). Hirata et al. observed also no significant effect of nicotine on mitochondrial dynamics in SH-SY5Y cells, resulting in the hypothesis that undifferentiated cells behave differently than already developed cells in response to nicotine treatment (Hirata et al. 2016). However, results obtained in hippocampal neurons indicated that nicotine affects mitochondrial dynamics by inducing mitochondrial fission in a Drp1-dependent manner (Godoy et al. 2018).

With respect to mitochondrial biogenesis in hippocampal neurons, the effect of nicotine was determined by analysis of PGC-1α expression, which is a transcription factor involved in mitochondrial biogenesis. Nicotine treatment at concentrations ranging from 5 to 25 μM increased the level of PGC-1α by about 50% relative to control cells, indicating increased mitochondrial biogenesis and suggesting an increase in the mitochondrial population. Experiments with the nAChR inhibitors α-bungarotoxin and dihydro-β-erythroidine implied that the observed nicotine effects were mediated by both α7-nAChR and α4β2-nAChR (Godoy et al. 2018).

Taken together, different results concerning changes in mitochondrial dynamics following nicotine treatment may relate to the different cell types used, as well as nicotine concentration and incubation time, all of which need to be taken into consideration when comparing those results. Nicotine effects on mitochondrial dynamics and turnover were addressed only by a few studies. Changes in mitochondrial dynamics appear to be at least partially a consequence of a nicotine effect on intracellular calcium homeostasis. Apart from that, modulation of mitochondrial network morphology and mitochondrial biogenesis is an important part of cellular responses to mitochondrial stress. Thus, taking into account the inhibitory effect of nicotine on the respiratory chain as well as its capability to induce oxidative stress, the consequences of nicotine exposure for mitochondrial dynamics and turnover should be also expected. Therefore, further investigation of these aspects of nicotine action in the cells would be highly interesting.

## Nicotine and mitochondria in cellular adaptation

Nicotine affects many aspects of mitochondrial physiology, as reviewed in the preceding sections. Despite this knowledge, the processes of cellular adaptation to long-term nicotine exposure, particularly in the context of mitochondrial function, are still poorly understood.

Prolonged nicotine exposure elicits nAChR up-regulation, which does not correlate with a change of mRNA levels, pointing to a post-transcriptional effect of nicotine. Indeed, nicotine was shown to up-regulate different nAChR subtypes via intracellular action on early maturation steps of ER-located species (Sallette et al. 2005). Moreover, nicotine exposure increases the ratio of mitochondrial versus non-mitochondrial nAChRs *in vivo*, but makes mitochondrial receptors less susceptible to the effects of specific ligands (Uspenska et al. 2017). Activation of nAChRs triggers intracellular signaling cascades leading to increased cell survival by attenuation of mitochondrial apoptosis (Akaike et al. 2010). This kind of interaction is responsible for the neuroprotective effect against β-amyloid-induced neurotoxicity in SH-SY5Y cells through the nicotine-mediated up-regulation of anti-apoptotic proteins Bcl-2, Bcl-xL and Mcl-1 (Xue et al. 2014). The anti-apoptotic effect of nicotine is not limited to neuronal cells, but it is also observed in normal and cancer cells of peripheral tissues (Deng 2014). This phenomenon is critically important in the context of developing resistance to chemotherapy, particularly in lung cancers. Nicotine prevented cisplatin- and etoposide-mediated reduction of Δ_Ψ_, activation of caspase-9 and translocation of Bax protein to mitochondria in human A549 lung carcinoma cells (Zhang et al. 2009). Phosphorylation of the pro-apoptotic protein Bad and up-regulation of the anti-apoptotic protein XIAP were mediated by the Akt signaling pathway. Moreover, experiments with cells lacking mtDNA (A549 ρ^0^ cells) revealed that intact mitochondria and proper mitochondrial function are critical for mediating the anti-apoptotic effects of nicotine (Zhang et al. 2009). Similarly, activation of α7-nAChR by nicotine inhibited cisplatin-induced mitochondrial-dependent caspase-3 activation and cytochrome c release in Raw264.7 and El4 cells. The up-regulation of the anti-apoptotic proteins Bcl-2 and Mcl-1 in these cells was also mediated by the PI_3_K-Akt signaling pathway (Wang et al. 2014). It was recently shown that regulation of cell survival by nicotine is realized by multiple nAChR subtypes localized on the OMM, which can control various pathways leading to induction of intrinsic, mitochondrial apoptosis (Lykhmus et al. 2014). Activation of these mitochondrial receptors may inhibit opening of the mPTP and the release of pro-apoptotic factors. It seems that the pro-survival effect of nicotine is a sum of the simultaneous activation of nAChRs located on the cell plasma membrane and on the OMM (Chernyavsky et al. 2015a; Skok et al. 2016).

As a amphiphilic compound, nicotine may easily penetrate cell membranes and directly interact with complexes of the mitochondrial electron transport system regulating their activity independently of nAChRs (Cormier et al. 2001). Chronic nicotine treatment led to an increase in malate and succinate dehydrogenases in rat frontoparietal cortex (Turégano et al. 2001) and in the mitochondrial antioxidant enzyme GST in rat brain (Bhagwat et al. 1998). A number of genes encoding components of the electron transport chain such as cytochrome c oxidase subunit I, cytochrome b and mitochondrial NADH dehydrogenase 4 were significantly modulated by nicotine in multiple rat brain regions. The differential expression of these genes in particular brain regions may be associated with different energy demands and reaction mechanisms in response to nicotine (Wang et al. 2009).

Regulation of multiple intracellular signaling pathways by chronic exposure to nicotine may lead to changes in electron transport chain activity, modulation of mitochondrial function like calcium buffering or ROS generation and inhibition of the intrinsic apoptosis pathway. Overall, the current evidence indicates an involvement of mitochondria in nicotine-mediated adaptive changes leading to cell survival.

## Summary and comments

**Fig. 1:**
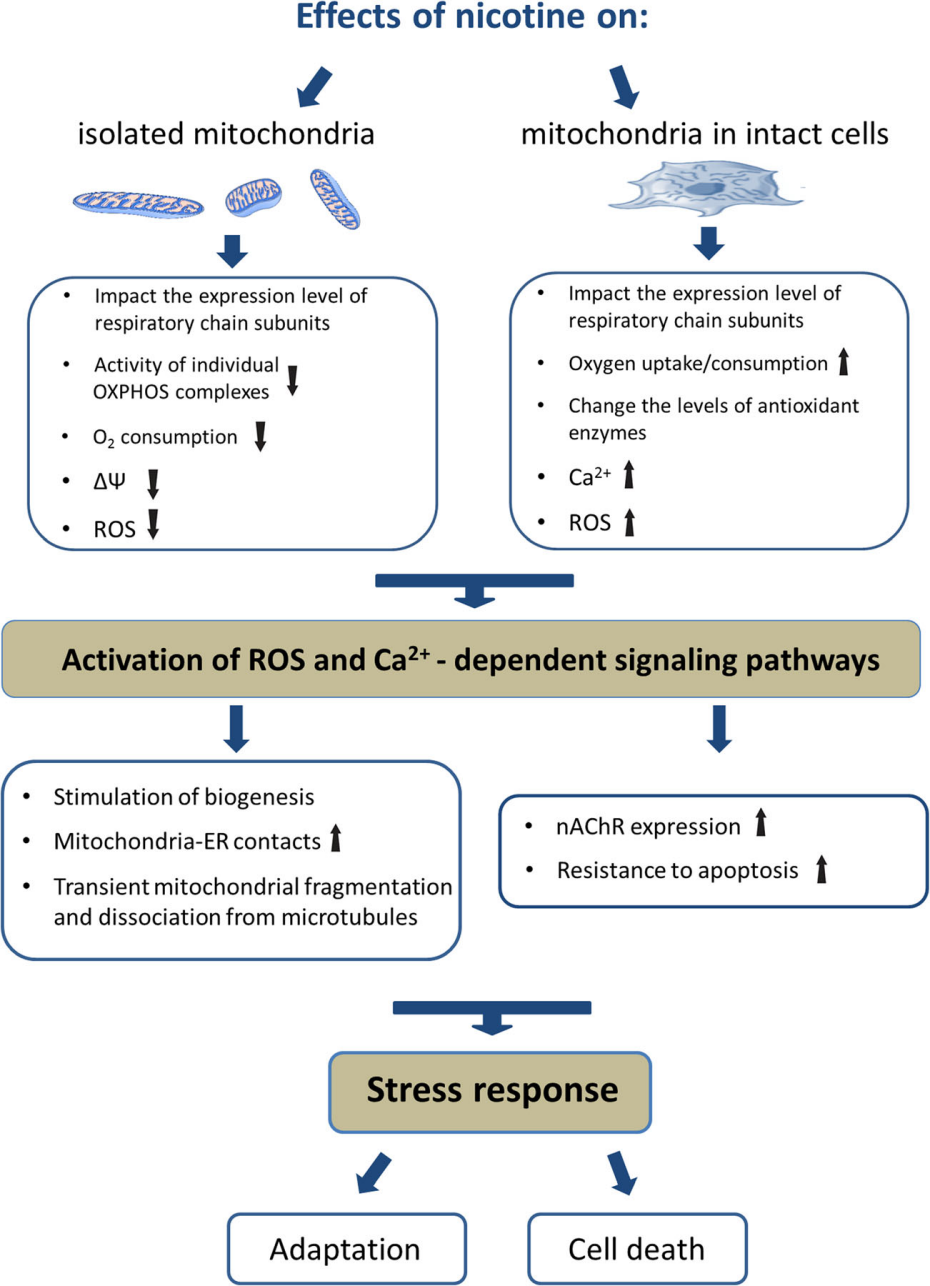
Summary of experimental evidence regarding the effects of nicotine on mitochondrial physiology from *in vitro* (isolated mitochondria) and *in situ* (mitochondria in intact cells) studies

The reviewed studies describe deleterious as well as beneficial effects of nicotine on mitochondrial and cellular function (Fig. 1).

The impact of nicotine on mitochondrial function has been studied in different experimental systems, such as isolated mitochondria, intact cells and animal models. Respiratory complex I and mitochondrial nAChR are suggested targets of direct nicotine action in mitochondria. Moreover, nicotine decreased susceptibility of mitochondria to mPTP opening, modulated mitochondrial network morphology and mitochondrial-ER associations. In some experimental models, nicotine increased intracellular ROS in a mitochondrial-dependent manner. Overall, however, the evidence indicates that remodeling of mitochondrial function by nicotine can influence cell and tissue physiology in multiple ways and these effects can be both detrimental as well as beneficial.

One of the common problems of most experimental models is the selection of nicotine dose, route of administration and treatment duration to closely mimic nicotine exposure of cells and tissues seen in smokers. Studies measuring nicotine concentrations in smokers report plasma levels of around 100-200 nM (Abu-Awwad et al. 2016). Thus, such concentrations should be considered physiological for studies with cultured cells. On the other hand, it is known that the sensitivity of different cells to applied drugs can strongly vary, particularly between primary cultures and stable cell lines. Such an effect was observed also in the case of nicotine: in the study by Zanetti et al., apoptosis induction in primary alveolar cells was observed at 100 nM nicotine, whereas 10 μM nicotine was needed to exert a similar effect in the mouse MLE-12 lung epithelial cell line, and human BEAS-2B bronchial epithelial cells appeared to be even more resistant to nicotine (Zanetti et al. 2014). This complicates the interpretation of some results obtained with cultured cells. Nevertheless, cell culture models are very useful for evaluating the mechanisms underlying the effects observed in animal models and in human studies.

In turn, in the case of animal studies, the main concern, besides dose selection, is the nicotine administration method. The majority of studies used intraperitoneal or subcutaneous injections, while reports of nicotine inhalation were much rarer (Phillips et al. 2015; Phillips et al. 2017; Werley et al. 2016). In rats receiving 1 mg/kg nicotine via intraperitoneal injection, sub-micromolar concentrations were detected in the plasma after 1 h, while nicotine concentrations dropped below 100 nM after 4 h, mainly due to its metabolism to cotinine (Jung et al. 1999). Nose-only inhalation as a method of nicotine administration (6.6 mg/kg daily, 13 weeks of exposure) resulted in plasma nicotine concentrations of about 2.5 μM in male and 3.5 μM in female rats ( Phillips et al. 2017). As for in vitro studies, the effects of doses of nicotine that are more closely mimicking the physiological dose range observed in human after exposure to nicotine containing products should also be further investigated in animal studies.

In conclusion, the effect of nicotine on mitochondrial physiology is carefully studied, but the nature of participation of nicotine in the mitochondrial function is still not resolved and understood. The coming few years should see the elucidation of this problem and of the character and mechanism of the action of nicotine on mitochondria.
